# Epidemiology of Leptospirosis: The First Literature Review of the Neglected Disease in the Middle East

**DOI:** 10.3390/tropicalmed7100260

**Published:** 2022-09-24

**Authors:** Elena Harran, Christo Hilan, Zouheira Djelouadji, Florence Ayral

**Affiliations:** 1 Laboratoire des Leptospires et d’Analyses Vétérinaires, Université de Lyon, VetAgro Sup, USC 1233, 69280 Marcy l’Etoile, France; 2 Faculty of Arts and Sciences, Holy Spirit University of Kaslik (USEK), Jounieh P.O. Box 446, Lebanon

**Keywords:** *Leptospira*, one health, diagnosis, epidemiology, middle eastern countries

## Abstract

Leptospirosis is a major zoonotic disease that has emerged worldwide, and numerous studies performed in affected countries have provided epidemiological knowledge of the disease. However, currently, there is inadequate knowledge of leptospirosis in the Middle East. Therefore, we grouped publications from various Middle Eastern countries to acquire a general knowledge of the epidemiological situation of leptospirosis and provide an initial description of the leptospiral relative risk and circulating serogroups. We conducted a detailed literature search of existing studies describing *Leptospira* prevalence and seroprevalence in Middle Eastern countries. The search was performed using online PubMed and ScienceDirect databases. One hundred and one articles were included in this review. Some countries, including Iran, Turkey, and Egypt, reported more publications compared to others, such as Lebanon, Kuwait, and Saudi Arabia. Frequently, the seroprevalence of leptospirosis varied considerably between and within countries. The prevalence of leptospirosis was comparable in most Middle Eastern countries; however, it varied between some countries. The methods of detection also varied among studies, with the microscopic agglutination test used most commonly. Some hosts were more recurrent compared with others. This review summarizes the epidemiological situation of *Leptospira* infection in the Middle East, reporting predominant serogroups—Sejroe, Grippotyphosa, Icterohaemorrhagiae, Autumnalis, and Pomona—that were identified in the most commonly tested hosts. Our findings emphasize the need to develop a deeper understanding of the epidemiology of *Leptospira* spp. and prioritize the disease as a public health problem in this region. To achieve this goal, increased awareness is critical, and more publications related to the topic and following a standardized approach are needed.

## 1. Introduction

Leptospirosis is a zoonosis that is prevalent worldwide and has major impacts on both humans and animals [1,2]. The disease is caused by species of *Leptospira*, a spirochaete bacterium with increasing genetic diversity [3,4]. To date, 38 species of pathogenic *Leptospira* have been described, and new species are continually being discovered [5]. The morbidity and mortality rates of leptospirosis in humans are estimated at 1 million and 60,000 cases, respectively [6]. Of the reported cases, 2.90 million disability-adjusted life years are estimated to be lost per annum [7]. Various human-acquired syndromes, ranging from flu-like to life-threatening hepatorenal syndromes, have been described to be associated with leptospirosis in the literature [8,9,10]. In severe cases, mortality rates vary between 5% and 20% [6]. Humans, as well as wild and domestic animals, can be infected either directly following contact with the body fluid of *Leptospira*-infected animals or indirectly when exposed to environments contaminated by leptospirosis [11,12,13,14]. Animal *Leptospira* infection presents not only as acute clinical manifestations observed in humans but also as a chronic infection that can lead to major economic depletion due to reproductive failure [2]. Following several human leptospirosis outbreaks reported worldwide [15], this infectious disease has been categorized as a (re-) emerging disease and is still qualified as such by the World Health Organization (WHO) [16].

The limit of understanding of the natural history of *Leptospira* infection and the under-recognition of its burden are due to insufficient diagnosis, largely as a result of the two stages experienced by the host [17,18]. The first stage of leptospirosis is the septicemic or acute stage, which occurs in the first week of infection, wherein the host generally shows symptoms of *Leptospira* circulating in the bloodstream [1,2,17,19]. The second stage of the disease is the immune stage, which generally occurs in the second week of infection, wherein the host starts to acquire and show anti-*Leptospira* antibodies in the serum [1,2,17,19]. Currently, the most accurate test to detect the acute phase of infection is the polymerase chain reaction (PCR) [18]; this technique is highly sensitive and can rapidly detect the *Leptospira* species [17,20]. Treatment following a positive PCR result at this stage might be effective, unlike the culture and microscopic agglutination test (MAT), which is less advantageous for early diagnosis. Culture is time-consuming and known for its difficulty in isolating *Leptospira*, whereas the MAT only detects antibodies indicating a past or current infection [8,21]. Nonetheless, MAT is considered the immunological reference standard method for leptospirosis experimental diagnosis by the World Organization for Animal Health (WOAH) [22] and the WHO [23]. Another supportive immunological test for the detection of antibodies is enzyme-linked immunosorbent assay (ELISA) [24]. Although the diagnostic accuracy of ELISA has not been completely established [25], the facilities of performing ELISA (manipulation with killed antigens) rather than MAT (live antigens) shows a promising alternative to several laboratories in tropical countries which reported its high sensitivity and specificity [26,27].

In recent years, cases of human and animal leptospirosis have been reported in numerous countries in the Middle East through direct and/or indirect diagnostic techniques. Human cases commonly involve farmers, rice field workers [28,29,30,31,32,33,34,35,36], travelers [37], and plumbers [38]. Leptospirosis cases in children and/or adults in contact with infected livestock or contaminated water have also been reported [28,39,40,41]. However, cases without a clear history of pathogenicity [42] and those without obvious occupational activities known to be risk factors for leptospirosis transmission have also been noted in some Middle Eastern countries and are qualified as “inner-city related”. Moreover, cases of co-infection with both dengue fever and leptospirosis have been described in the Middle East [43]. Animal case reports normally include numerous species but mainly involve livestock. Furthermore, the direct detection of leptospirosis in water resources has been reported [44]. Preventive measures to reduce health and economic consequences following *Leptospira* infection in the community relies on a deep understanding of the epidemiology, public awareness, and vaccination of domestic animals and populations at risk. The knowledge of the predominant serogroup of a host is an important guide for an effective vaccination since the latter strategy only bestows a protective immunity restricted to homologous or closely associated serovars [45].

This review aimed to acquire general knowledge about the epidemiological situation of leptospirosis in the Middle East and provide an initial description of the leptospiral relative risk and circulating serogroups in this region to develop and adopt prophylactic strategies if necessary. This study focused on collecting and revising the available data on the prevalence of *Leptospira* in humans and animals in the Middle East. We expect to determine the variability in *Leptospira* spp. prevalence and seroprevalence in different Middle Eastern countries according to variations in related records/publications. Variability was expected because leptospirosis outbreaks are related to local factors, such as environmental and meteorological factors, and because it is commonly reported in reviews from different geographical areas, such as Africa, the Pacific Islands, and China. For instance, Africa reported a variability in the seroprevalence between 2.3% and 19.8% in hospital patients having febrile illness [46]; in Pacific Islands (y = number of islands), the seroprevalence varied between 19.6% and 45.0% in cattle (y = 4) and between 10% and 88% in humans (y = 7) [47], and in China, human case studies reported a seroprevalence between 8.2% and 56.7% [48]. In addition, we expected to find similar genetic profiles or circulating serogroups in particular hosts, such as humans, cattle, and rodents of the Middle East, as has been reported in other areas. For instance, we expect to find the predominance of the serogroups Icterohaemorrhagiae (ICT) in humans and rodents [49,50] and Sejroe (SJ) in cattle [51], as has been shown in previous literature. However, for some other hosts, no specific serogroups were expected.

## 2. Materials and Methods

### 2.1. Literature Search

We conducted a detailed literature search of the existing studies describing the *Leptospira* seroprevalence and prevalence in Middle Eastern countries. In our review, we included the following countries; Bahrain, Cyprus, Egypt, Iran, Iraq, Jordan, Kuwait, Lebanon, Oman, Palestine, Qatar, Saudi Arabia, the Syrian Arab Republic, Turkey, the United Arab Emirates, and Yemen.

The search was conducted using PubMed and ScienceDirect online databases. Search terms were used to manually find relevant articles and included “((Leptospira) OR (Leptospirosis)) AND (Middle East) AND (Prevalence)”, “((Leptospira) OR (Leptospirosis)) AND (Middle East country name) AND (Prevalence)”, “((Leptospira) OR (Leptospirosis)) AND (Middle East) AND (Seroprevalence)”, “((Leptospira) OR (Leptospirosis)) AND (Middle East country name) AND (Seroprevalence)”, “((Leptospira) OR (Leptospirosis)) AND (Middle East) AND (Human)”, “((Leptospira) OR (Leptospirosis)) AND (Middle East) AND (Animals)” without any stipulation or precondition on publication date. Similar research terms were also used in Google Scholar to extract relevant articles.

### 2.2. Inclusion and Exclusion Criteria

Both fully accessible publications and abstracts describing the seroprevalence and prevalence of leptospirosis in humans or animals were included in this review, regardless of their publication date. Publications that did not describe this topic were excluded. In addition, publications written in a language other than English were excluded; nevertheless, if the latter publication contained an English abstract with relevant data for the review, it was included.

### 2.3. Data Extraction

The data extracted from the retrieved publications, abstracts, or reviews included the author, year of publication, and geographical location. Additional information on the chosen hosts, their effectiveness, prevalence, seroprevalence, method of detection [culture, PCR, MAT, ELISA, serology for serogroup identification (SSI), or direct method], reference serovars, and identified serogroups were also extracted. Serogroups referring to any host in the Middle East were reported, regardless of the titers obtained in the SSI. The latter methods included the MAT, indirect immunofluorescent antibody test (IFAT), complement fixation test, lysis-agglutination test (LAT), immunoglobulin test, silver staining (SS), latex agglutination test, macroscopic plate agglutination test (MPAT), microtube agglutination analysis, and agglutination test.

### 2.4. Distribution of Publications

Information regarding the methods of detection applied and the serogroups identified were retrieved from both research papers and case studies because the objective was to dispose of all the available information regarding the circulating *Leptospira* in the Middle East. Nonetheless, information regarding the prevalence and seroprevalence of *Leptospira* was only retrieved from research papers. However, the cartography demonstrating the geographical distribution of the Middle East publications included all retrieved publications (research papers, case studies, and reviews).

## 3. Results

### 3.1. Literature Search

A database search using the previously cited queries retrieved 7169 articles (458 articles from PubMed and 6711 from Science Direct), of which 7058 were excluded. The exclusion criteria were based on the absence of adequate information about leptospirosis in humans or animals or those that were not published in English. The review yielded 111 articles (104 research papers and case studies and 7 reviews), with publication dates varying between 1947 and 2021. Seventeen of the latter articles were published prior to 2000; however, the remaining 94 articles were published after a remarkable increase in publication throughout the last decade.

### 3.2. Distribution of Leptospira Seroprevalence and Prevalence in Middle Eastern Countries

**Figure 1:**
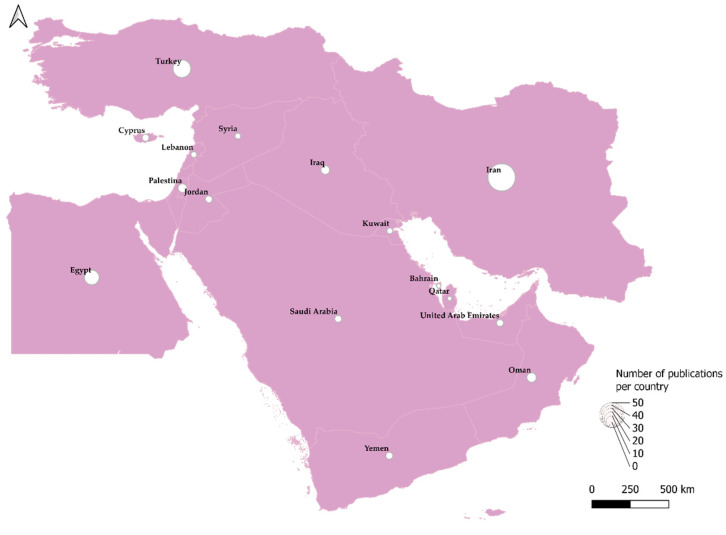
Geographical distribution of the Middle Eastern publications. Map created with DIVA-GIS version 7.5 and designed with QGIS 3.16.1 Hannover.

The reported publications comprised research papers, case studies, and review articles that included 14 countries (Figure 1). Publications related to Oman were included but did not record any seroprevalence or prevalence, as they only involved case studies [37,40,43]. Detailed data on prevalence, seroprevalence, and serogroup/serovar information related to each publication can be found in Table S1.

Some countries tested a high number of hosts but recorded a low seroprevalence and contrariwise. For example, Palestine tested 2018 rodents and Cyprus tested 261 cattle, 195 goats, and 507 sheep, and both the countries reported a seroprevalence of 0% [52,53,54] by SSI. However, Egypt tested two cats and two weasels, and one of each host showed positive antibody titers [55]. 

Studies from other countries have focused on determining the seroprevalence in particular hosts. For example, two studies from Jordan reported a seroprevalence of 49.7% and 92.3% in cattle only by SSI [56,57]. Among studies from the United Arab Emirates, one showed a seroprevalence of 4.1% [58] in camels, and another showed a seroprevalence of 1.7% in cattle [59]. In Yemen, a seroprevalence of 41.3% [60] and 42% [61] by ELISA was reported in humans, and a seroprevalence of 6.7% by SSI was reported in camels in Saudi Arabia [62].

The Syrian Arab Republic has one of the lowest rates of publications on this topic, with only one publication in 1984. However, the seroprevalence by SSI was reported as 2.9%, 11.9%, and 33.9% in sheep, goats, and cattle, respectively [63]. In contrast, Iran, Turkey, and Egypt were the most reported countries in the Middle East, with 50, 21, and 16 publications, respectively, with both low and high seroprevalence reported. A seroprevalence of 1.1% [64] and 0.05% [65] was reported by SSI in humans in Iran and Turkey, respectively. Again, using SSI, a seroprevalence of 0% [44,55,66,67] was reported in camels, cattle, donkeys, dogs, horses, rodents, and sheep in Egypt. A seroprevalence of 71% [68] in dogs, 82.1% [69] in humans, and 78.4% [70] in cattle were reported by SSI in Iran, Turkey, and Egypt, respectively.

Iran, Turkey, and Egypt also reported studies that estimated the prevalence of the *Leptospira* infection in various hosts, although many of these reported prevalence values were low. For example, a prevalence of 0% was reported in Iran [71] in goats, and a prevalence of 0% was reported in Egypt [44,55,66] in humans, buffaloes, camels, cattle, donkeys, horses, sheep, water buffaloes, and weasels by PCR and/or culture. Moreover, a prevalence of 1.4% [72] in cattle has been reported in Turkey. Only one remarkably high prevalence of 74.4% [69] was reported in humans in Turkey by dark field microscopy (DFM). The highest prevalence in the Middle East was reported in Iraq (94.3%) by DFM and direct microscopic examination (DFE) [73]. The detailed data on *Leptospira* seroprevalence and prevalence in Middle Eastern countries are shown in Table 1 and Table 2, respectively.

### 3.3. Surveillance Methods Used in the Middle East

Various direct or indirect methods of detection (MOD) have been reported in 104 publications, as shown in Table 3. The indirect methods—MAT or ELISA—were most commonly used, either solely (n = 52) with MAT in addition (n = 38), or in parallel with other methods (n = 40). Culture or direct PCR MODs were also commonly used either solely (n = 8) or in parallel with others (n = 26) but in fewer numbers of publications. Some of the reported MODs are LAT [66], IFAT [97], and MPAT [116] for SSI and immunoperoxidase (IP) [137], DFM [73], DFI [134], and DFE [120] for direct diagnosis. The PCR target genes varied according to each publication, and the list of genes is shown in Table 4.

### 3.4. Geographical Distribution of Serogroups in the Middle East 

**Figure 2:**
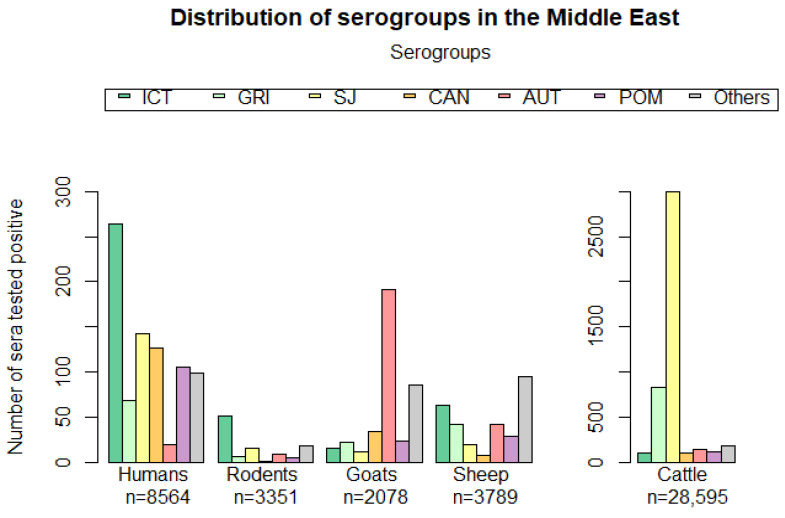
Distribution of serogroups in the five major hosts in the Middle East. Plot created by RStudio (RStudio Team (2021), RStudio: Integrated Development Environment for R; RStudio, PBC, Boston, MA, USA; URL http://www.rstudio.com/, accessed on 22 June 2022).

We relied on all SSI tests (such as MAT, LAT, IFAT, and MPAT) to demonstrate the serovars/serogroups circulating in most Middle Eastern countries. Reference serovars were also determined to evaluate the results in a dependent manner, as presented in Table 5. A total of 51 reference serovars, each referring to a specific serogroup, were reported in the Middle East. Twenty serogroups were identified, each with its own occurrence in the Middle East; however, only those related to the predominant hosts in this region are presented (Figure 2). The top five reported hosts in the Middle East were cattle, humans, rodents, sheep, and goats, with a population density of 28,595, 8564, 3351, 3789, and 2078, respectively. The predominant serogroups identified in the previously cited hosts were SJ (n = 3200), Grippotyphosa (GRIP) (n = 938), ICT (n = 515), Autumnalis (AUT) (n = 404), and Pomona (POM) (n = 102). Other serogroups such as Cynopteri (n = 12), Celledoni (n = 7), and Mini (n = 5) were also identified but had low numbers. However, each host had a predominant serogroup, including AUT for goats; SJ for cattle; and ICT for humans, sheep, and rodents. All serogroups presented in Table 5, except for Andamana, were identified in Egypt, Turkey, Iran, Iraq, Palestine, Jordan, and the Syrian Arab Republic. Other countries also reported serogroup identification in other hosts; however, they were not presented because the number of hosts was relatively low.

## 4. Discussion

To the best of our knowledge, this is the first review to summarize the prevalence and seroprevalence of leptospirosis and *Leptospira* infection in the Middle East. The disease, which continues to inflict a high burden worldwide, has been neglected in this particular region. From a broad perspective, almost all Middle Eastern regions reported information on the prevalence and/or seroprevalence of leptospirosis and *Leptospira* circulating serogroups. However, the prevalence values and serogroup distribution differed according to hosts and countries.

The prevalence and seroprevalence of leptospirosis in humans appear important in the Middle East, especially when compared with the prevalence and seroprevalence in other regions worldwide. Many leptospirosis outbreaks have been described in tropical and subtropical regions, including Latin America, Northern America, Southern Asia, and Africa, with some having incidence rates reaching 100 per 100,000 habitants per year [46,138,139]. However, to the best of our knowledge, a seroprevalence and prevalence of >41% have not been reported in these regions, despite having an adequate climate to support *Leptospira* survival and favorable human exposure. Indeed, the highest seroprevalence rates reported recently in Latin America were 40.2%, 23.6%, 8.8%, and 7.2% in Brazil, Peru, Colombia, and Ecuador, respectively [140]. In addition, the highest prevalence reported in Africa is 19.8% [46]. These results may be due to the sampling design being subject to selection bias by only reporting severe/laboratory-confirmed cases and/or hospital patients with acute febrile illness. Such a research design cannot provide an accurate presentation of leptospirosis cases because the disease is only known to cause severe complications in 5% to 10% of cases [141]. The reported prevalence and seroprevalence rates in the Middle East are higher (>42%) when either applying the same or different selection bias. This region not only reported acute cases but also asymptomatic cases relevant to the controlled group and recorded higher seroprevalence than the previously cited regions [84]. For instance, Iran tested seroprevalence in both healthy and hospitalized patients and recorded values of 48.5% and 64.7%, respectively [84,95]. Therefore, the risk factors for acquiring the disease in the Middle East may be more important than those in other regions.

SSI combined with MAT was reported in our review as the indirect method of serogroup identification. The tests related have the advantage of identifying a particular serogroup using available reference strains for manipulation. Therefore, the host serum is exposed to a panel of serovars, and the results vary in a dependent manner. Various serogroups have been reported in the Middle East; however, these serogroups are unlikely to be exhaustive, given that they could have been expanded with the use of additional reference serovars. In the top five hosts (humans, rodents, goats, sheep, and cattle) of this region, the predominant serogroup was SJ in cattle; AUT in goats; and ICT in humans, sheep, and rodents. The predominance of each serogroup in each of these hosts has been commonly reported in the literature, except in the case of goats. Serogroup SJ has been the most commonly reported serogroup in cattle in different countries worldwide, including the United States of America, France, Ireland, the Netherlands, Belgium, and others [51,142,143,144,145]. The serogroup ICT is commonly reported in rats worldwide [50,146] and is known to be the major causative agent of leptospirosis in humans [147,148]. In sheep, the predominance of the latter (ICT) serogroup supports previous observations in other countries [149]. In the case of goats, the predominance of the serogroup AUT has not been reported regularly in other countries worldwide [150,151,152] but has been commonly reported in related publications in the Middle East. However, this result appears robust given that the number of MAT positive cases associated with AUT (n = 191) in goats was considerably higher than the expected serogroup SJ (n = 12), which is considered predominant in goats in other countries [153,154]. Nonetheless, local variation may have led to such results and, as this is the first epidemiological study in the Middle East, it may be an indication of the most predominant serogroup in goats in this region. However, this assumption should be consolidated or proven by further studies. Moreover, the preponderance of the serogroup ICT in sheep in the Middle East can be explained by the high probability of their infection by rodents (carrying ICT) or their capacity for selective carriage of some ICT strains, as described for other hosts (pigs) in the literature [2]. The predominance of the serogroup ICT in humans in the Middle East may be due to infection by cattle, sheep, and rodent carriers of this serogroup, during their occupational work (farmers, rice farmers), travel, or contact with contaminated water. It may also be due to the importance of pathogenicity caused by the latter (ICT) serogroup leading to leptospirosis susceptibility and, therefore, its diagnostic examination [1]. Despite the various biases within the collected data, data regarding the serogroups in the Middle East remain informative because the objective of this review was to describe the circulating serogroups regardless of their titers and MODs used. In addition, no records of human or animal vaccination were mentioned in the selected publications; therefore, the serogroups detected in the Middle East were not concluded according to antibodies developed by vaccination but by infection. Such results help orient the type of vaccine that will be regarded as effective to each host. For instance, protection against *Leptospira* serovar ICT and SJ through vaccination should reduce the risk of leptospirosis in humans and cattle, respectively. Nonetheless, the adequacy of the serogroup repartition in the Middle East may be questioned because of the possibility of cross-reactions, which may lead to the consideration of serogroups that are not actually present. However, the distribution of the serogroups was analyzed at the whole population scale to minimize the effects of cross-reaction in our results, as has been performed in previous studies [144,155]. 

As expected, the reported seroprevalence and prevalence differed according to studies, likely due to variability in the MODs of *Leptospira* spp. Some diagnostic methods, such as PCR or any other direct method, can only detect nucleic acids in the first week of the host infection, known as the bacteremia phase of infection [1]. However, other MODs, such as MAT, ELISA, or any other SSI, can detect antibodies days after the onset of the disease and for a much longer duration [27]. The difference in the time margin between the persistence of the bacteria and the antibodies in host tissues lessens the chances of prevalence reports, in contrast to the chances of seroprevalence reports.

Several studies have reported null prevalence and seroprevalence. For some studies, this can be due to the small sampling size (<30 samples), 26 goats [71], 22 camels and 14 horses [44], five sheep [67], two weasels [55], one cattle, and one dog [67], which may not indicate the true distribution of the infection in the geographic location. Moreover, the sampling size may not be sufficient to detect an infected or exposed host if present in the population; indeed, a sampling size of 26 individuals allows the detection of a minimum prevalence or seroprevalence of 11% [156]. In the case of Cyprus, the country did not state human leptospirosis cases for several consecutive years [157], except for the year 2003 when 0.3 cases of 100,000 habitants were infected [158]. However, the case definition may not respond to sensitive detection of the *Leptospira* infection given that many asymptomatic or moderate cases could be experienced by the host, including humans. The European Center for Disease Prevention and Control reports were sent in accordance with the case definitions established by the European Union that included Cyprus. The general principles for the application of the case definitions are to only report laboratory-confirmed symptomatic cases, while suspected cases were only regarded as cases if they revealed a clear clinical picture with a judicious laboratory diagnosis [157,158]. In addition, the 2008 case definition was restricted to pathogenic *Leptospira* spp., namely *L. interrogans*, whereas, starting in 2012, all pathogenic *Leptospira* spp. were considered in the detection panel [157]. Such pathogenic *Leptospira* species restrictions may lead to an underestimation of the incidence [158]. Moreover, a null seroprevalence was reported in cattle, goats, and sheep in Cyprus, and, to the best of our knowledge, only a few imported calves tested positive in 1983 [54]. However, one publication is insufficient to determine the seroprevalence of ruminants in Cyprus but could explain the possibility of acquiring such seroprevalence. Surprisingly, studies in Palestine reported a null seroprevalence in rodents, even with a high sampling number. This may have been due to the antibody response of the *Leptospira*-infected rodent, which is frequently found to be under the threshold of positivity [159,160]. It may also be due to the remarkable variation in prevalence and/or seroprevalence from one rat colony to another starting from 0%, as reported in previous studies [161,162]. Thus, the apparent seroprevalence in Palestine could be underestimated compared with the true seroprevalence of the tested rodents because the sampling concerns a limited number of colonies (n = 2). Although the seroprevalence is null in rodents, infections have been detected in Palestine in both humans (seroprevalence of 1.9%) [52] and cattle (seroprevalence of 8.5% and 9.5%) [52,53]. The apparent seroprevalence in both hosts was elucidated by co-authors, who suggested that numerous outbreaks of leptospirosis in hundreds of cattle were the cause of human infection, such as those reported a few years prior to sampling [163,164].

Some countries have reported a remarkable seroprevalence range in the human population. The greater the number of studies combining various factors, such as the time interval between the two studies, the spatial variation, and the design of the study adapted in different publications related to the same country, the greater the seroprevalence variability.

An important time interval in the same country could lead to variations in disease epidemiology. For example, in Egypt, a seroprevalence of 0.5% was reported in 1957 [63], whereas a seroprevalence of 49.7% was reported in 2015 [44]. The risk factors for acquiring the disease depend on the environmental features and animal carrier abundance, which differ with the spatial variation, explaining such variability [165,166]. Therefore, spatial variability in the same country with a large surface area and an important distribution of studies throughout those areas, such as Iran, Turkey, and Egypt, could lead to seroprevalence variability. In addition, the lack of comparable design studies in the same host may lead to variable seroprevalence. The design of the study is specific to each publication because the sampling criteria were unique; some groups were chosen because of their tendency to be infected due to their occupations or professional activities, some were chosen randomly as controlled groups [65,66], and others were sampled for a differential diagnosis (cases of hepatitis, acute febrile illness for humans, and brucellosis for cattle) [74,76,127]. However, when the same type of group was chosen, low seroprevalence variability was observed. For instance, in the case of humans in Yemen, when only two publications targeting similar types of groups (people at risk) were reported, the seroprevalence ranged between 41.3% [60] and 42% [61]. The sampling period may also have had a major impact on the incidence of leptospirosis throughout the year because of the seasonal pattern of the disease and its recrudescence in specific seasons, in which the highest incidence occurs mainly in summer or/and fall in temperate countries and in rainy seasons in warm-climate regions [8]. Therefore, the seroprevalence can differ throughout the year in a particular country and within the same population. For instance, a study in the north of Iran (temperate region) demonstrated a higher prevalence of leptospirosis in individuals in autumn and summer compared with that in spring [83]. In summary, the greater the number of studies combining the latter factors, the greater the seroprevalence variability. Moreover, the more these factors vary between publications, the lower the comparability of the prevalence or seroprevalence.

The epidemiological knowledge of leptospirosis is unclear for some countries in the Middle East because of the type and content of publications. For instance, studies in Oman only reported case studies [37,40,43]; therefore, the magnitude of leptospirosis in Oman remains unknown. However, a high prevalence is expected due to the globalization of travel and trade, occupational activities, and the temperate climate of the country [167]. Other countries, such as Saudi Arabia and the United Arab Emirates, only reported a prevalence and/or a seroprevalence in a few hosts (camels and cattle) due to a greater interest in describing the health status of the mammals that are largely present in these countries. Epidemiological knowledge is also lacking in some countries in the Middle East due to the absence of studies. For instance, in the twenty-first century, Lebanon, Kuwait, and the Syrian Arab Republic did not renew their interest in studying *Leptospira* infection. This suggests that leptospirosis is not within the public health policy priorities and/or that its burden is underestimated; this is in contrast to that noted in other Middle Eastern countries, such as Iran, which continuously show their interest in studying the disease by attempting to revise and authenticate its detection methods [168,169].

The time interval between the reported publications, the difference in the spatial environment, the particular design of the study adapted, and the number of publications related to each country led to the cognizance of leptospirosis variable epidemiology in the Middle East. Therefore, heterogeneous strategies applied in each country and between different Middle Eastern countries should be limited as much as possible, and a harmonized strategy should be adapted for better comparison of epidemiological studies relating to the seroprevalence of leptospiral infection.

- For the detection methods, PCR and culture should be prioritized for direct detection and MAT and ELISA for indirect detection. These methods can be applied in parallel when sampling particular hosts, whereas their efficacy can be limited to others. For instance, direct methods should be prioritized in the case of rodents because they have a low antibody response to leptospiral infection [159].
- As MAT remains the reference detection method, a minimum and common panel of serovars from selected serogroups should be included in all Middle Eastern countries that require shared reference strains. The minimum number of serogroups that should be tested are ICT, GRIP, SJ, CAN, AUT, and POM.
- A common human case definition should be a reference to all Middle Eastern countries to report the maximum, confirmed, and suspected number of clinical cases of *Leptospira*. Random sampling could be performed to describe the epidemiological situation in humans more comprehensively, considering asymptomatic or moderate cases.
- For the surveillance of *Leptospira* infection in domestic animals, an analysis of data on a continuous basis following diagnostic examinations in veterinary laboratories should be considered to determine the distribution of *Leptospira*. Such data should be communicated to the organizations and the public, indicating the applied diagnostic method of examination. Moreover, the sampling modalities should be stated by the community of veterinary practitioners in order for them to be interpreted at the Middle East region level.
- Veterinary practitioners should be encouraged to provide all available information on animals and herds to enable a good diagnosis and improve epidemiological analyses. Information on the reason for examination, and the farms, herds (size and type), and animals (age, sex, clinical status) at diagnostic testing will facilitate improved epidemiological analysis and the ability to suggest risk factors to move toward more efficient risk-based surveillance in the future.

## 5. Conclusions

This review summarizes the epidemiological situation of *Leptospira* infection in the Middle East. Leptospirosis was found to be endemic in the countries of the Middle East for which data were available, excluding Cyprus. Variability in the prevalence and seroprevalence of *Leptospira* spp. has been reported in these countries. Furthermore, several serogroups have been reported in hosts in the Middle East, including ICT, GRIP, SJ, CAN, AUT, and POM. Some serogroups might be considered for certain hosts, whereas others are commonly reported in hosts worldwide. Therefore, a deeper understanding of the epidemiology of *Leptospira* spp. is required. In addition, leptospirosis should be prioritized as a public health problem in this region, for which increased awareness is critical. Therefore, more publications following a harmonized and appropriate study design, while also prioritizing particular seasons leading to leptospirosis recrudescence, and specific spatial environment or risk factors favorable for bacterial existence and survival are needed to achieve this goal.

## Figures and Tables

**Table 1:** Summary of *Leptospira* seroprevalence in the Middle East according to the test performed (SSI or ELISA) and the country from which the sampling originated.

Country	Number of Articles	Publication Year	Host	Total Number of Host Tested	Seroprevalence Range SSI	Total Number of Host Tested	Seroprevalence Range Elisa	References
Cyprus	1	2000	Cattle	261	0%	-	-	[54]
			Goats	195	0%	-	-	[54]
			Sheep	507	0%	-	-	[54]
Egypt	14	1957–2018	Human	4334	0.5–49.7%	1278	16%	[44,66,67,74,75,76,77,78]
			Buffaloes	409	15.4–30%	97	20%	[44,55,75,79]
			Cats	2	50%	-	-	[55]
			Camels	82	0–50%	-	-	[44,75,79,80]
			Cattle	1093	0–78.4%	-	-	[44,55,66,67,70,75,79]
			Donkeys	73	0–29%	-	-	[44,55,67,75]
			Dogs	394	0–58.3%	-	-	[44,55,66,67,81]
			Goats	317	1.5–42.1%	-	-	[66,67,75]
			Horses	14	0%	-	-	[44]
			Pigs	158	14.3–43.8%	-	-	[67,75]
			Rodents	1087	0–75.9%	-	-	[44,55,66,67]
			Sheep	736	0–45.4%	-	-	[44,66,67,75,79,82]
			Water buffaloes	99	14.1%	-	-	[66]
			Weasels	2	50.00%	-	-	[55]
Iran	36	2003–2021	Human	1782	1.1–61.6%	3983	10.4–64.7%	[34,64,83,84,85,86,87,88,89,90,91,92,93,94,95,96,97]
			Buffaloes	130	25.40%	-	-	[98]
			Caspian seal	164	5.3–25.8%	-	-	[99,100]
			Cats	213	4.9–27.03%	-	-	[101,102]
			Cattle	1178	17.4–37.8%	-	-	[103,104,105,106,107]
			Dogs	48	71%	-	-	[68]
			Donkeys	170	40–41.2%	-	-	[108,109]
			Goats	210	8.5–11.7%	-	-	[110,111,112]
			Horses	541	7.8–39.2%	-	-	[108,109,113]
			Mules	20	39.2%	-	-	[109]
			Rodents	187	21.2–33%	-	-	[114,115]
			Sheep	412	10.9–19.3%	-	-	[105,110]
Iraq	4	2010–2017	Cattle	334	57.3–85%	96	7.3%	[73,116,117]
			Dogs	218	17%	-	-	[118]
			Goats	153	22.4–57.9%	-	-	[116]
			Sheep	199	24.6–42.9%	-	-	[116]
Jordan	2	1992–2019	Cattle	448	49.7%	240	92.3%	[56,57]
Palestine	2	1948	Human	207	1.9%	-	-	[52]
			Cattle	1665	8.5–9.5%	-	-	[52,53]
			Rodents	2018	0%	-	-	[52,53]
Saudi Arabia	1	2009	Camels	90	6.7%	-	-	[62]
Syrian Arabic Republic	1	1984	Human	407	0%	-	-	[63]
			Cattle	1894	33.9%	-	-	[63]
Turkey	14	1999–2016	Sheep	1735	2.9%	-	-	[63]
			Goats	1203	11.9%	-	-	[63]
			Horses	258	0%	-	-	[63]
			Human	1834	0.05–62.8%	192	42.8–82.1%	[65,69,119,120,121,122]
			Rodents	59	8.5%	-	-	[123]
			Cattle	21722	3.4–45%	4560	14–38.6%	[72,119,124,125,126,127]
			Dogs	116	44%	-	-	[128]
			Sheep	200	8%	-	-	[119]
			Buffaloes	93	32.3%	-	-	[129]
United Arab Emirates	2	1994–2020	Camels	73	4.1%	-	-	[58]
			Cattle	-	-	350	1.7%	[59]
Yemen	2	2015–2018	Human	-	-	467	41.3–42%	[60,61]

**Table 2:** Summary of *Leptospira* prevalence in the Middle East according to the test performed (PCR or culture) and the country from which the sampling originated.

Country	Number of Articles	Publication Year	Host	Total Number of Host Tested	Prevalence Range PCR	Total Number Of Host Tested	Prevalence Range Culture	References
Egypt	5	1957–2015	Human	175	0%	175	0%	[44]
			Buffaloes	38	0%	38	0%	[44,55]
			Cats	2	50%	2	50%	[55]
			Camels	22	0%	22	0%	[44]
			Cattle	634	0–1.1%	634	0–1.1%	[44,55]
			Donkeys	30	0%	30	0%	[44,55]
			Dogs	193	11.3–12%	261	0–11.3%	[44,55,81]
			Horses	14	0%	14	0%	[44]
			Rodents	370	24–26%	1461	0.37–7%	[44,55,66]
			Sheep	124	0%	189	0%	[44,55,82]
			Water buffaloes	12	0%	12	0%	[66]
			Weasels	2	0%	2	0%	[55]
Iran	9	2010–2018	Human	119	50.4%	-	-	[97]
			Camels	130	14.6%	-	-	[130]
			Caspian Seals	128	18.7–20.3%	-	-	[100]
			Cats	132	21.2%	-	-	[131]
			Cattle	238	14.3–43%	98	1.0%	[71,103,132]
			Goats	26	0%	-	-	[71]
			Rodents	151	3.3–11.3%	151	0%	[115]
			Sheep	110	4.5%	-	-	[71]
Iraq	1	2017	Dogs	37	13.5%	-	-	[118]
Kuwait	1	1983	Rodents	-	-	49	16.3%	[133]
Lebanon	1	1947	Rodents	-	-	70	5.70%	[134]
Turkey	6	2006–2015	Human	-	-	192	14–45.7%	[69,120]
			Cattle	499	0–9.4%	-	-	[72,135]
			Rodents	102	16.9–46.5%	-	-	[123,136]
Saudi Arabia	1	2009	Camels	-	-	36	0%	[62]

**Table 3:** Methods of detection performed in 104 publications (research articles and case studies) in the Middle East.

Major Tests Performed	Number of Articles	Supplementary Information
MAT only	38	
ELISA only	14	
PCR only	7	mPCR, qPCR, nPCR
Culture only	2	
MAT, ELISA *	8	
MAT, culture * †	3	
MAT, PCR * †	2	
ELISA, PCR * †	1	
PCR, culture †	1	
ELISA, culture * †	4	
MAT, ELISA, PCR * †	1	
MAT and others *	3	DFE, IFAT, MPAT
Culture and others * †	3	DFE, CFT, LAT, DFI, IFT, IGT
PCR and others †	1	IFAT, S
Culture, PCR and others * †	1	IFAT
MAT, culture and PCR * †	2	
MAT, culture, PCR and others * †	1	PGE
MAT, culture and others * †	4	IFAT, DFM, SS, DFE, LAG, MPAT
Culture, ELISA, and others * †	1	DFM, DFE
MAT, ELISA, culture and others * †	1	MAA, DFM
Others *	5	AT, LAT, IFA
Others	1	IP
	104	

mPCR, multiplex PCR; qPCR: quantitative PCR; nPCR, nested PCR; DFE, dark field examination; IFAT, indirect immunofluorescent antibody test; CFT, complement fixation test; LAT, lysis-agglutination test; DFI, dark field illumination; IFT, Immunofluorescent technique; IGT, immunoglobulin test; S, sequencing; PGE, Pulsed-gel electrophoresis; DFM, dark field microscopy; SS, silver staining; LAG, latex agglutination test; MPAT, macroscopic plate agglutination test; MAA, Microtube agglutination analysis; AT, Agglutination Test; IP, immunoperoxidase; *, MAT and ELISA in parallel with other techniques; †, PCR and culture in parallel with other technics.

**Table 4:** PCR target genes used by 15 studies.

PCR Target Genes	Number of Articles
rrs (16s rRNA)	6
lipl32	1
rrs (16s rRNA) and lipl32	1
g1/g2 primers	2
lig1/lig2 primers	1
g1/g2 primers and lig1/lig2 primers	1
flaB	1
hap1	1
NM	1
	15

NM, not mentioned.

**Table 5:** Reference serogroups and serovars in the Middle East.

S *	S	S *	S	S *	S	S *	S	S *	S
AND	Andamana	BAT	Bataviae		Icterohaemorrhagiae		Pyrogenes		Wolffi
AUS	Australis	CAN	Benjamin		Mankarso	SEM	Patoc	TAR	Hyos
	Bratislava		Canicola	JAV	Javanica		Semaranga		Tarassovi
	Jalna	CEL	Celledoni		Poi	SJ	Balcanica		
AUT	Autumnalis	CYN	Cynopteri		Sorexjalna		Bovis*		
	Bulgarica	DJA	Djasiman	MINI	Georgia		Burgas		
	Butembo		Sentot		Mini		Hardjo		
	Rachmat	GRI	Grippotyphosa		Swajizak		Istrica		
BAL	Arborea	HEB	Borincana	PAN	Panama		Polonica		
	Ballum		Hebdomadis *	POM	Pomona		Saxkoebing		
	Castellonis	ICT	Copenhageni	PYR	Alexi		Sejroe		

AND, Andamana; AUS, Australis, AUT, Autumnalis, BAL, Ballum, BAT, Bataviae, CAN, Canicola, CEL, Celledoni, CYN, Cynopteri, DJA, Djasiman; GRI, Grippotyphosa, HEB, Hebdomadis, ICT, Icterohaemorrhagiae, JAV, Javanica, PAN, Panama; PYR, Pyrogenes, SEM, Semaranga, SJ, Sejroe, TAR, Tarassovi, S *, serogroup, S, Serovar, *: Bovine strain of Leptospira.

## Data Availability

The data presented in this study are available in Supplementary Materials.

## Supplementary Materials

The following supporting information can be downloaded at: https://www.mdpi.com/article/10.3390/tropicalmed7100260/s1, Table S1: Summary of *Leptospira* prevalence, seroprevalence and serovars distribution in the Middle East.
